# Allgrove Syndrome in Adults: A Case of an Atypical Presentation

**DOI:** 10.7759/cureus.98638

**Published:** 2025-12-07

**Authors:** Meryem Maizi, Fatima Belabbes, Hanane Delsa, Saloua Elamari, Imane Ben Elbarhdadi

**Affiliations:** 1 Gastroenterology and Hepatology, Cheikh Khalifa International University Hospital, Mohammed VI University of Sciences and Health, Casablanca, MAR; 2 Research Unit, Mohammed VI Center for Research and Innovation, Rabat, MAR; 3 Endocrinology, Diabetology, Metabolic Disease, and Nutrition, Cheikh Khalifa International University Hospital, Mohammed VI University of Sciences and Health, Casablanca, MAR

**Keywords:** achalasia, adrenal insufficiency, adult, alacrima, allgrove syndrome, delayed diagnosis

## Abstract

Allgrove syndrome, or "Triple A" syndrome, is a rare autosomal recessive disorder characterized by achalasia, alacrima, and adrenal insufficiency. Typically diagnosed in childhood or adolescence, its adult presentation remains under-recognized and challenging. We report the case of a 29-year-old Moroccan woman with chronic dysphagia, who was ultimately diagnosed with Allgrove syndrome following the discovery of type 1 achalasia, primary adrenal insufficiency, and alacrima. The diagnosis was delayed, and symptoms had persisted for over a decade before appropriate evaluation. Clinical, endoscopic, radiologic, and manometric findings confirmed the diagnosis. Therapeutic management included corticosteroids and pneumatic dilation, with a favorable outcome. This case highlights the importance of considering Allgrove syndrome in adults with unexplained dysphagia and systemic symptoms.

## Introduction

Allgrove syndrome, also known as “Triple A” syndrome, is a rare autosomal recessive disorder characterized by a triad of achalasia, alacrima, and adrenocorticotropic hormone (ACTH)-resistant adrenal insufficiency [1,2]. Although most cases are identified during childhood or adolescence, adult presentations are rare and often manifest with atypical features, notably neurological involvement alongside the classic symptoms [3].

We report the case of a 29-year-old woman with Allgrove syndrome in whom the diagnosis was significantly delayed because of the insidious progression of long-standing, unexplored dysphagia. This case underscores the diagnostic challenges associated with adult-onset forms of the condition.

## Case presentation

A 29-year-old Moroccan woman, born to consanguineous parents (first-degree cousins), presented with a 13-year history of progressively worsening dysphagia. Her childhood history was notable for a foreign body impaction (a coin) that required endoscopic removal, along with chronic anemia of unknown etiology. Intermittent dysphagia began at the age of 16 and gradually progressed over the years.

She reported postprandial vomiting, nocturnal gastroesophageal reflux, unintentional weight loss, chronic fatigue, dry cough, blurred vision, and joint pain. Neurological symptoms included rhinolalia (nasal speech), orthostatic lightheadedness, and episodic dysarthria. Family members had observed generalized skin hyperpigmentation and melanonychia over the past decade.

**Figure 1:**
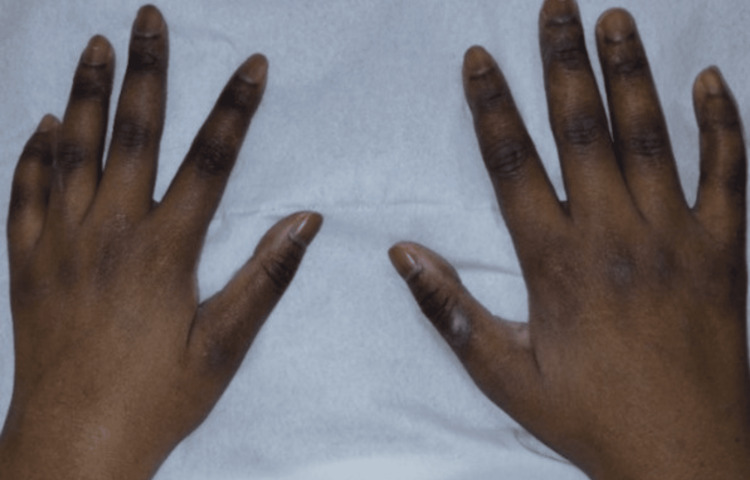
Marked melanoderma of both hands

On physical examination, blood pressure measurements were within the normal range, and her body mass index (BMI) was 16.9 kg/m². She appeared clinically dehydrated and exhibited diffuse cutaneous and mucosal hyperpigmentation, particularly in the skin folds (Figure 1). 

**Figure 2:**
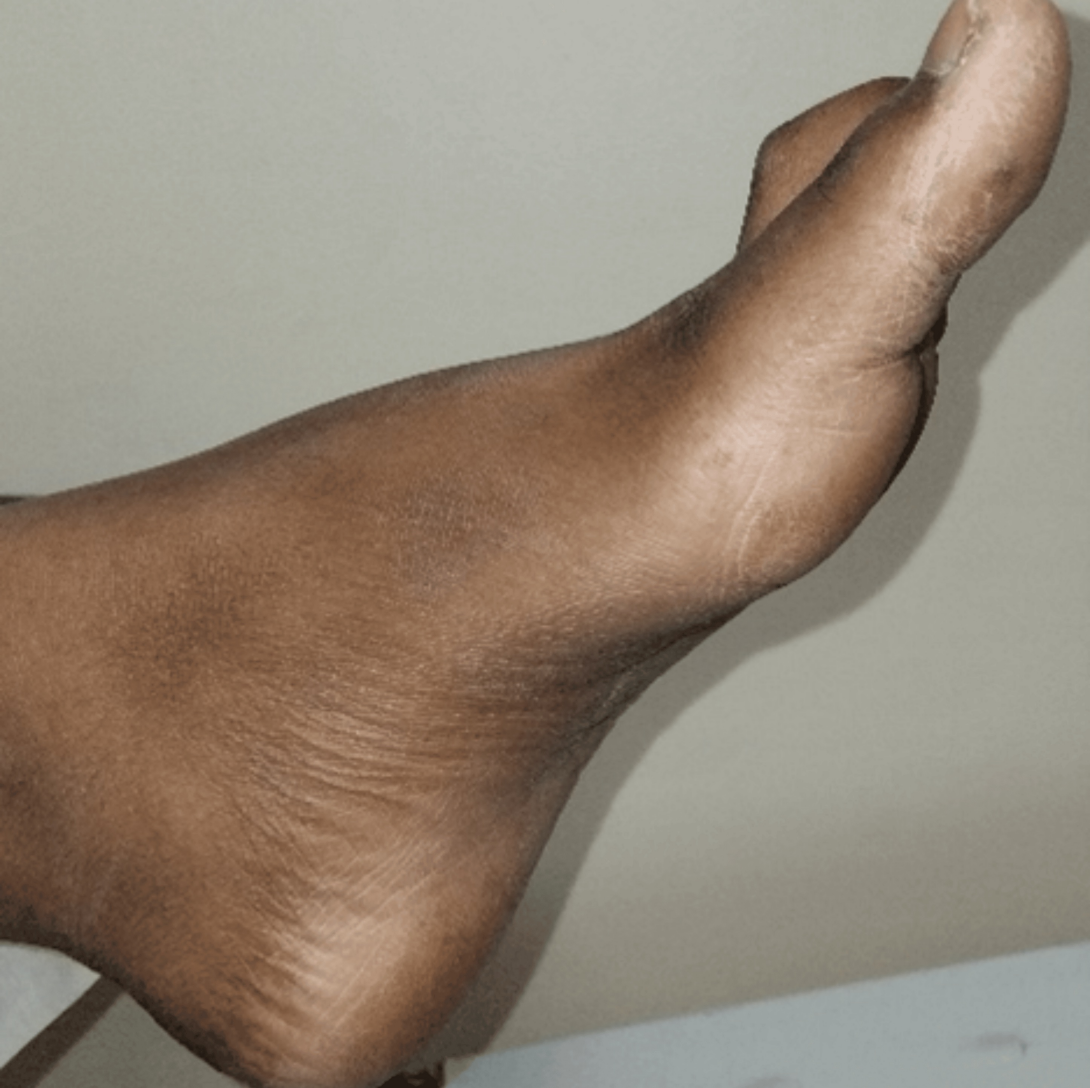
Marked concavity of the plantar surface of the foot

Neurological examination revealed rhinolalia, mildly slowed speech, and increased deep tendon reflexes in the lower extremities. Bilateral pes cavus was also noted (Figure 2).

Ophthalmologic evaluation showed decreased tear production in the right eye, with a positive Schirmer’s test (13 mm) [4]. Both eyes were examined, and excavation of the optic papillae was observed bilaterally. 

Laboratory investigations (Table 1) revealed a markedly reduced morning serum cortisol level (0.3 µg/dL) accompanied by a significantly elevated ACTH concentration (3593 pg/mL), confirming the diagnosis of ACTH-resistant adrenal insufficiency. No ACTH stimulation test was performed, as the combination of clinical features and biochemical findings was considered sufficient to establish the diagnosis. Anti-21 hydroxylase antibodies were negative, ruling out autoimmune Addison’s disease. Mild hypokalemia (3.2 mmol/L) was observed, while serum sodium levels remained within normal limits, which was discussed in the context of isolated glucocorticoid deficiency. A normocytic, normochromic anemia was present (hemoglobin 9.9 g/dL) with a low reticulocyte count (43.2 x 10⁹/L). Normal serum ferritin and elevated vitamin B12 levels (907 pg/mL) were noted. 

**Table 1:** Lab findings in our patient ACTH: adrenocorticotropic hormone

Lab Finding	Result	Reference Range	Unit
Morning serum cortisol at 8:00 a.m	0.3	4 - 20	µg/dL
ACTH	3593	4.7 - 48.8	pg/mL
Anti-21-hydroxylase antibodies	0	< 0.4	u/ml
Serum potassium (K⁺)	3.2	3.5 - 5.1	mmol/L
Serum sodium (Na⁺)	140	136 - 145	mmol/L
Hemoglobin	9.9	11.5 - 17.5	g/dL
Mean corpuscular volume	85.3	76 - 96	fl
Reticulocyte count	43.2	75 - 120	× 10⁹/L
Serum ferritin	56	20 - 200	ng/ml
Vitamin B12	907	187 - 893	pg/mL

**Figure 3:**
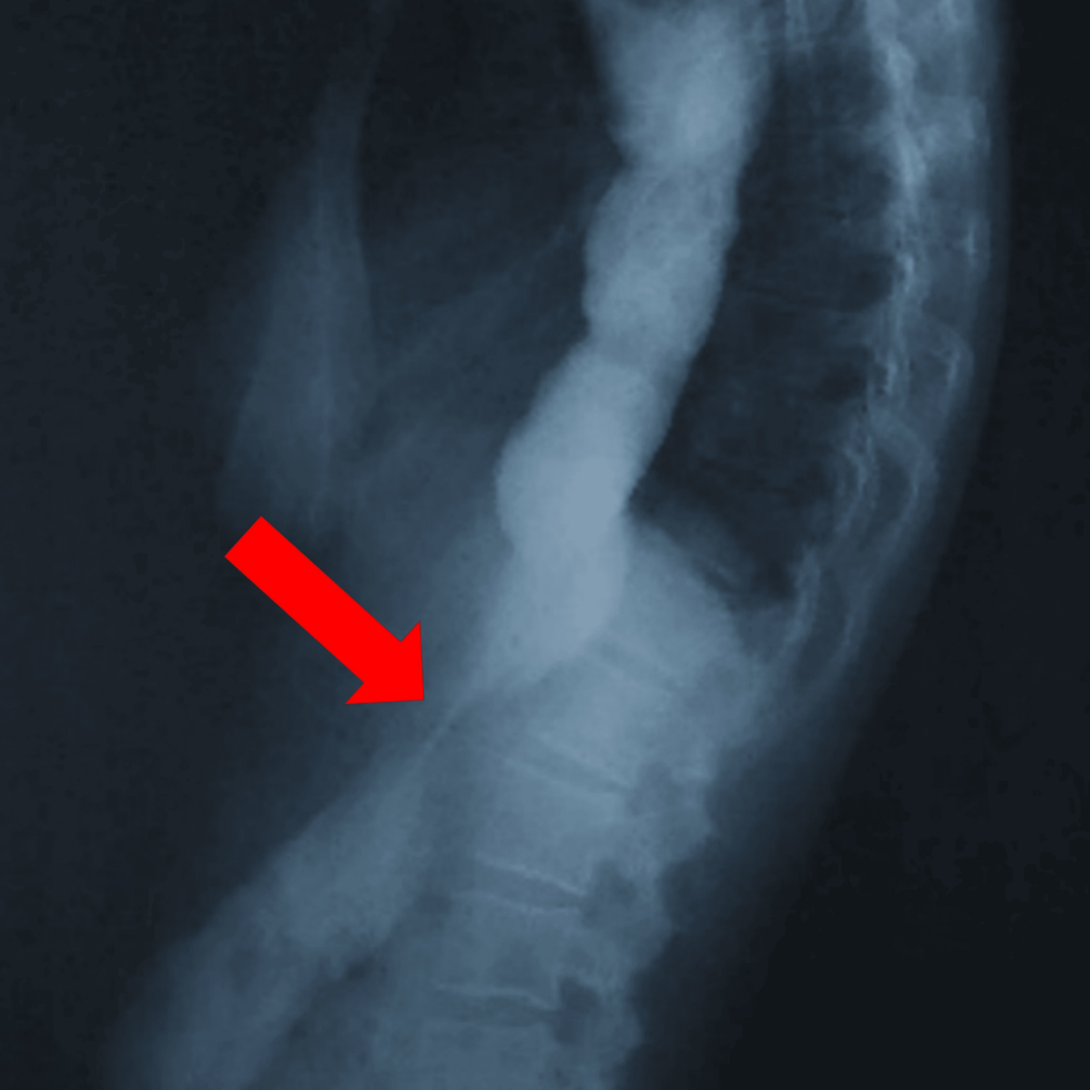
Barium swallow study showing the classic “bird’s beak” appearance, characterized by tapering of the distal esophagus with proximal dilation (red arrow)

Upper gastrointestinal endoscopy showed a markedly dilated esophagus with retained food material and increased resistance at the lower esophageal sphincter. A barium swallow demonstrated the classic “bird’s beak” appearance with tapering at the gastroesophageal junction (Figure 3). 

A thoracoabdominal CT scan revealed significant esophageal dilation without mural thickening or extrinsic compression. The adrenal glands appeared morphologically normal, a finding that supports primary adrenal insufficiency due to ACTH resistance rather than destructive adrenal disease.

High-resolution esophageal manometry was performed in the upright position. The integrated relaxation pressure (IRP) was 21.7 mmHg, slightly below the upper normal limit of 22.0 mmHg, and the IRP normal upper limit in the upright position is 15.0 mmHg. Importantly, 100% of peristalsis was absent. This profile, in conjunction with endoscopic findings of a dilated esophagus with resistance at the gastroesophageal junction, is consistent with type I achalasia (“classic” achalasia) according to the Chicago Classification v4.0 [5].

The diagnosis of Allgrove syndrome was strongly suspected based on the coexistence of type I achalasia confirmed by manometry, primary adrenal insufficiency demonstrated by hormonal testing, and clinical alacrima, despite the unavailability of genetic testing due to financial constraints. 

The patient was initially managed with intravenous methylprednisolone (40 mg/day), which was subsequently transitioned to oral hydrocortisone at 60 mg/day, with a plan for tapering and adjustment according to clinical response. Mineralocorticoid replacement (fludrocortisone) was considered but deemed unnecessary due to normal blood pressure and serum electrolytes. For achalasia, pneumatic balloon dilation (35 mm) was performed under fluoroscopic guidance as the first session, with good tolerance; future sessions or alternative therapies (peroral endoscopic myotomy (POEM) or Heller myotomy) will be considered if symptoms recur. Symptomatic management of alacrima was initiated with regular use of preservative-free artificial tears to prevent ocular surface complications. 

At the one-month follow-up, the patient reported substantial clinical improvement, including complete resolution of dysphagia, partial regression of skin hyperpigmentation, and relief of ocular dryness. She demonstrated good adherence to corticosteroid therapy, and no adverse events were reported.

She is under ongoing follow-up. Seven months after the initial intervention, dysphagia remained well controlled, and the patient continues to be monitored for adrenal function, esophageal symptoms, and ocular health.

## Discussion

Since its first description in 1978, fewer than 200 cases of Allgrove syndrome have been reported worldwide, with adult-onset presentations remaining rare and only occasionally described [6]. The case presented above highlights the importance of increased awareness of this syndrome to enable earlier diagnosis. Here, we provide a review of the current literature on this still poorly understood disorder.

The “Triple A” syndrome is a rare genetic disorder characterized by the triad of achalasia, adrenal insufficiency, and alacrima [1]. It is most commonly diagnosed in childhood or adolescence and is rarely identified in adults, as illustrated in our case.

Allgrove syndrome is inherited in an autosomal recessive pattern and results from mutations in the AAAS gene located on chromosome 12q13, which encodes the ALADIN protein involved in nucleocytoplasmic transport. Over 60 different mutations have been identified, explaining the phenotypic variability observed [7]. A founder mutation, c.1331+1G>A, is frequent in North Africa, particularly reported in Moroccan, Tunisian, Algerian, and Libyan patients [2,8].

Dysfunction of the ALADIN protein disrupts the transport of specific proteins through the nuclear pore complex, impairing cellular function in the adrenal glands, esophagus, and lacrimal glands, thus explaining the multisystemic involvement seen in this syndrome [9].

The classic clinical presentation includes the following triad: progressive dysphagia of functional appearance, confirmed as achalasia by esophageal manometry; slowly progressive peripheral adrenal insufficiency (Addison’s disease), suggested by severe physical asthenia and orthostatic hypotension, confirmed by low cortisol, elevated ACTH, negative anti-21 hydroxylase antibodies, and morphologically normal adrenal glands on imaging; and alacrima, manifesting as ocular dryness that can be complicated by keratitis [10,11].

Notably, neurological manifestations are frequently associated, particularly cranial nerve involvement (notably IX and X), which control the function of the soft palate and may explain the nasal speech (rhinolalia) often found in adult patients with this syndrome, as seen in our patient. Symmetrical distal peripheral neuropathy (sensory, motor, or mixed) and hypotonia with altered deep tendon reflexes, which can be decreased, absent, or rarely increased (as in our case), have also been reported. Neurological and autonomic dysfunctions are more prevalent in adult patients [3]. These features may be related to dysregulation of cortisol-sensitive neuronal genes due to oxidative stress [7]. Some authors have proposed the term “Quadruple A syndrome” instead of the classical “Triple A” to account for these neurological symptoms [12]. 

The diagnosis is based on clinical evidence of the triad, supported by functional tests (lacrimal test, hormonal assays, and esophageal manometry), and confirmed by identification of AAAS gene mutations. In our case, genetic testing was not performed due to financial constraints [13].

Management of Allgrove syndrome is multidisciplinary and symptomatic, including endoscopic or surgical treatment of achalasia, esophageal dilation, POEM, or Heller’s myotomy; lifelong glucocorticoid replacement therapy for adrenal insufficiency; and regular ocular care to prevent complications related to alacrima [10].

This poorly understood syndrome offers multiple avenues for research, notably in the precise identification of the many implicated genetic mutations, a better understanding of ALADIN’s role in cellular oxidative stress, and its contribution to multisystem organ involvement [14].

## Conclusions

Allgrove syndrome is a rare autosomal recessive disorder, usually diagnosed in childhood, with adult-onset cases being uncommon and often atypical. Our patient illustrates the diagnostic challenges in adulthood, where long-standing dysphagia, subtle alacrima, and hyperpigmentation may be overlooked.

Neurological features such as rhinolalia, pes cavus, or hyperreflexia can provide important diagnostic clues. Early recognition is essential, as timely endocrine replacement and achalasia management can prevent adrenal crises and severe nutritional deficits. Management is inherently multidisciplinary, requiring coordination between endocrinology, gastroenterology, ophthalmology, and neurology. The considerable genotypic and phenotypic variability underscores the need for further research, particularly in precision medicine and gene therapy.
